# Supplementary material for: Vaccine Hesitancy among Parents and Its Association with the Uptake of Measles Vaccine in Urban Settings in Khartoum State, Sudan
Source: Vaccines (Basel). 2022 Jan 28;10(2):205. doi: 10.3390/vaccines10020205 (PMC8875338; doi:10.3390/vaccines10020205)
Supplement: Supplementary file 1 [file vaccines-10-00205-s001.zip › vaccines-1528007-supplementary.pdf]

Supplementary Materials

Table S1. Frequency distribution of the 15 PACV items (n = 495).

No.	PACV's items		N (%)
1	Have you ever delayed having your child get measles vaccine for reasons other than illness or allergy?	Yes	89 (18.0)
		No	403 (81.4)
		I don't know	3 (0.6)
2	Have you ever decided not to have your child get measles vaccine for reasons other than illness or allergy?	Yes	11 (2.2)
		No	481 (97.2)
		I don't know	3 (0.6)
3	How sure are you that following the recommended measles vaccine schedule is a good idea for your child?	0-5	15 (3.0)
		6-7	42 (8.5)
		8-10	438 (88.4)
4	Children get more shots of measles vaccine than are good for them.	Strongly agree	16 (3.2)
		Agree	20 (4.0)
		Not sure	16 (3.2)
		Disagree	293 (59.2)
		Strongly disagree	149 (30.1)
		Missing	1 (0.2)
5	I believe that measles is a severe disease.	Strongly agree	306 (61.8)
		Agree	171 (34.5)
		Not sure	5 (1.0)
		Disagree	13 (2.6)
6	It is better for my child to develop immunity by getting sick than to get a shot.	Strongly agree	25 (5.1)
		Agree	12 (2.4)
		Not sure	2 (0.4)
		Disagree	222 (44.8)
		Strongly disagree	234 (47.3)

7	It is better for children to get fewer vaccines at the same time.	Strongly agree	56 (11.3)
		Agree	58 (11.7)
		Not sure	13 (2.6)
		Disagree	251 (50.7)
		Strongly disagree	117 (23.6)
8	How concerned are you that your child might have a serious side effect from a shot of measles vaccine?	Not at all concerned	206 (41.6)
		Not concerned	190 (38.4)
		Not sure	5 (1.0)
		Concerned	60 (12.1)
		Very concerned	34 (6.9)
9	How concerned are you that any one of the measles vaccine shots might not be safe?	Not at all concerned	251 (50.7)
		Not concerned	162 (32.7)
		Not sure	18 (3.6)
		Concerned	48 (9.7)
		Very concerned	16 (3.2)
10	How concerned are you that a shot of measles vaccine might not prevent measles?	Not at all concerned	209 (42.2)
		Not concerned	187 (37.8)
		Not sure	17 (3.4)
		Concerned	61 (12.3)
		Very concerned	20 (4.0)
		Missing	1 (0.2)
11	If you had another infant today, would you want him/her to get all the recommended (measles) shots?	Yes	483 (97.6)
		No	8 (1.6)
		I don't know	4 (0.8)
12	Overall, how hesitant about measles vaccine shots would you consider yourself to be?	Not at all hesitant	283 (57.2)
		Not hesitant	192 (38.8)
		Not sure	3 (0.6)
		Hesitant	12 (2.4)
		Very hesitant	5 (1.0)

13	I trust the information I receive about measles vaccine shots.	Strongly agree	241 (48.7)
		Agree	237 (47.9)
		Not sure	7 (1.4)
		Disagree	8 (1.6)
		Strongly disagree	1 (0.2)
		Missing	1 (0.2)
14	I am able to openly discuss my concerns about shots with my child's doctor.	Strongly agree	269 (54.3)
		Agree	202 (40.8)
		Not sure	11 (2.2)
		Disagree	11 (2.2)
		Strongly disagree	2 (0.4)
15	All things considered, how much do you trust your child’s doctor?	0-5	25 (5.0)
		6-7	34 (6.9)
		8-10	436 (88.0)
